# An autopsy case of ovarian mucinous cystic tumor complicated by ovarian abscess and a review of the English literature

**DOI:** 10.1002/ccr3.6507

**Published:** 2022-11-16

**Authors:** Daisuke Suzuki, Shiori Meguro, Koji Inagaki, Masahiro Hashimoto, Hideya Kawasaki, Isao Kosugi, Yasunori Enomoto, Miho Sugiyama, Mayu Fukushima, Toshihide Iwashita

**Affiliations:** ^1^ Division of Diagnostic Pathology Chutouen General Hospital Kakegawa Japan; ^2^ Department of Regenerative and Infectious Pathology Hamamatsu University School of Medicine Hamamatsu Japan; ^3^ Division of Nephrology Chutouen General Hospital Kakegawa Japan; ^4^ Division of Radiology Chutouen General Hospital Kakegawa Japan; ^5^ Institute for NanoSuit Research, Preeminent Medical Photonics Education & Research Center Hamamatsu University School of Medicine Hamamatsu Japan; ^6^ Division of Diagnostic Pathology Hamamatsu University School of Medicine Hamamatsu Japan

**Keywords:** autopsy, ovarian abscess, ovarian mucinous cystic tumor

## Abstract

Ovarian tumors are rarely associated with abscesses. Herein, an autopsy case of an ovarian mucinous cystic tumor complicated by an abscess, along with a review of previous cases, suggests the necessity of considering ovarian abscess as a cause of inflammation in patients with the ovarian tumors.

## Graphical abstract

Cases of ovarian tumors complicated by abscesses are extremely rare and have not been reviewed previously. This autopsy case report, along with the literature review, is useful in raising awareness of ovarian tumors complicated by abscesses.

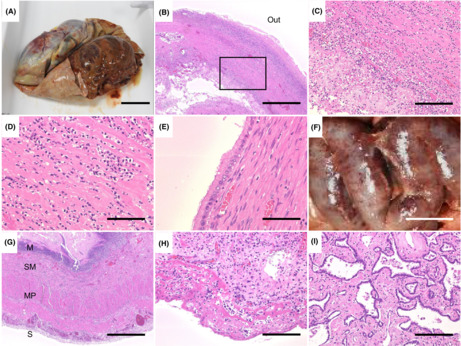

## 1 INTRODUCTION

Ovarian abscesses usually occur following an infection of the fallopian tubes and commonly develop as tubo‐ovarian abscesses (TOAs). TOA is an advanced pelvic inflammatory disease (PID), which often occurs in premenopausal women in their 30s and 40s.^1^ Ovarian abscesses are caused by several aerobic and anaerobic microorganisms such as gram‐negative bacilli (*Bacteroides* spp. and *Escherichia coli*) and *Streptococcus* spp. (*S. viridans* and *S. agalactiae*). The ascending route of infection is the most common. Although TOA tends to occur before menopause, postmenopausal TOA accounts for 6%–18% of all TOAs.^2^ TOAs can occur secondary to intra‐abdominal lesions such as appendicitis^3^ and diverticulitis.^4^, ^5^ It is very rare for ovarian tumors to be associated with ovarian abscesses, and approximately 20 cases have been reported to date.^6^, ^7^, ^8^, ^9^, ^10^, ^11^, ^12^, ^13^, ^14^, ^15^, ^16^, ^17^, ^18^, ^19^, ^20^, ^21^, ^22^, ^23^

In this article, we report a case of an ovarian mucinous cystic tumor with an abscess in an elderly woman. The cause of fever, leukocytosis, and elevated C‐reactive protein (CRP) level was thought to be pneumonia at the time of admission; however, autopsy examination revealed that it was due to an ovarian tumor complicated by an abscess. In addition to the case presentation, a review of published cases of ovarian abscesses complicated by the ovarian tumors has been discussed.

## 2 CASE HISTORY

**FIGURE 1:**
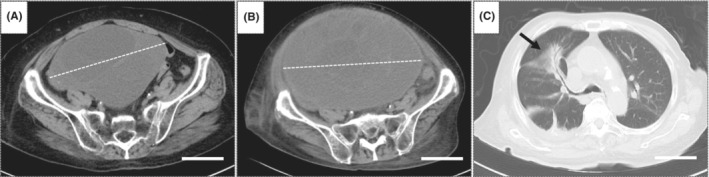
Diagnostic imaging on admission. (A) A non‐contrast computed tomography (CT) scan of the abdomen 15 months prior to admission shows a cystic tumor of the ovary, 19 cm in diameter. The white dotted line indicates the diameter of the tumor. (B) A non‐contrast CT scan of the abdomen at admission shows a cystic tumor of the ovary, 24 cm in diameter. The white dotted line indicates the diameter of the tumor. (C) The arrow indicates a high‐density area along the bronchus in the upper lobe of the right lung in the non‐contrast CT scan of the chest at admission. Scale bars = 5 cm (A–C).

An 82‐year‐old woman undergoing hemodialysis for chronic kidney disease caused by diabetes mellitus was admitted to our hospital with fever and diarrhea. She had previously been diagnosed with a right ovarian mucinous cystic tumor (5 years ago: 8 cm in diameter; 15 months ago: 19 cm in diameter) using abdominal computed tomography (CT) (Figure 1A). It was managed conservatively because she refused to undergo surgery. On admission, her lower abdomen was distended; however, she did not complain of abdominal tenderness. Her body temperature, pulse rate, respiration rate, and blood pressure were 37.5°C, 105/min, regular, 26 breaths/min, and 150/70 mmHg, respectively. The abdomen was soft; the liver and spleen were not palpable, and a soft mass was palpated in the lower abdomen. Routine blood tests were as follows: hemoglobin, 10.1 mg/dl; white blood cell count, 13,000/mm^3^, with 90% neutrophils; and platelet count, 235,000/mm^3^. *The oxygen saturation* of the peripheral artery (room air) was 93%. Blood biochemical studies showed urea nitrogen, 48.2 mg/dl; creatinine, 6.4 mg/dl; glucose, 113 mg/dl; total protein; 6.1 mg/dl, albumin, 2.4 mg/dl; and CRP, 26.35 mg/dl. Blood culture results were negative. Non‐contrast abdominal CT revealed a right ovarian mucinous cystic tumor (24 cm in diameter) (Figure 1B). Chest radiography showed an infiltrative shadow in the upper lobe of the right lung, which was confirmed on chest non‐contrast CT (Figure 1C). The non‐contrast chest CT suggested that the lesion in the right upper lobe could be community‐acquired pneumonia, neoplastic lesion, vasculitis, granulomatous disease, or hemorrhage. Of the diseases listed earlier, the possibility of community‐acquired pneumonia was considered based on its frequency and blood test results showing inflammation, such as high‐CRP levels. The ovarian tumor had gradually increased in size over the past 5 years. Imaging studies during that time showed that the ovarian tumor was not malignant, and therefore, the lung lesion was not considered to be metastatic from the ovarian tumor.

She was treated with ceftriaxone, a broad‐spectrum antibiotic often used as the initial treatment for community‐acquired pneumonia. During hospitalization, the blood cultures were negative. However, the white blood cell count remained high at 22,800/mm^3^, with 90% neutrophils and a CRP level of 21.73 mg/dl on the 5th day of hospitalization, implying that the antibiotics were not effective. As her general condition deteriorated, she was not given aggressive treatment and she died 8 days after admission.

**FIGURE 2:**
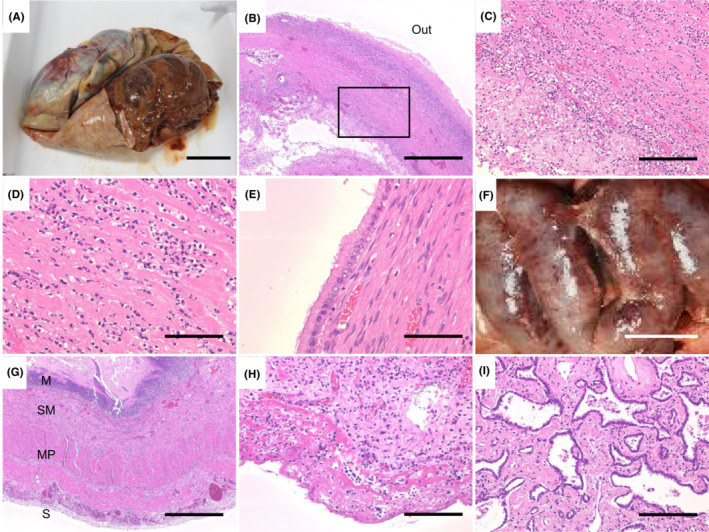
Gross and histological photographs of the ovary, ileum, and lung at autopsy. (A) The right ovary that was removed shows a cystic tumor. The pus had already been removed. (B) The ovarian cyst wall was observed at low magnification (×40). The epithelium covering the cyst was detached, showing fibrin deposition and neutrophil infiltration. ‘Out’ indicates the outside of the tumor. (C) The rectangle corresponds to the area shown in (B). The cyst wall was infiltrated with numerous neutrophils (×200). (D) The cyst wall was infiltrated with numerous neutrophils, but there was no obvious necrosis (×400). (E) The cytoplasm of the remaining cuboidal epithelium was filled with mucus (×400). (F) The ileum adherent to the ovarian tumor. Macroscopically, erythema was prominent, suggesting the presence of a high degree of inflammation. (G) Histologically, there was a high degree of transmural inflammatory cell infiltration of the ileal wall (×40). M, SM, MP, and S indicate mucosal layer, submucosal layer, muscularis propria, and serosa, respectively. (H) High degree of neutrophilic infiltration in the serous and subserosal layers (×400). (I) Well‐differentiated adenocarcinoma with little or no invasion was seen in the upper lobe of the right lung (×200). Scale bars = 5 cm (A and F), 200 μm (B and G), 40 μm (C and I), and 20 μm (D, E, and H).

An autopsy was performed 5 h after death. The right ovarian tumor occupied the pelvic and lower abdominal cavity and compressed the ileum. The tumor was adherent to the surrounding organs, especially the ileum and the abdominal wall. A small amount of ascites was observed. The tumor weighed approximately 5 kg. The incision of the tumor revealed a multifocal cyst containing 3.5 L of viscous, reddish‐brown, and pus‐like fluid flowing from the ovary (Figure 2A). *Escherichia coli*, *Bacteroides vulgatus*, and *Staphylococcus epidermidis* were cultured from the ovarian contents. Histologically, the ovarian tumor wall showed dense neutrophil infiltration and abscesses (Figure 2B–D). Most epithelial cells had detached from the wall due to the inflammation; however, the remaining epithelium consisted of columnar cells with mucus in the cytoplasm (Figure 2E), without invasion. Since the detachment of the epithelium was caused by inflammation, it was difficult to differentiate whether the tumor was benign or borderline. Thus, the ovarian tumor was diagnosed as a mucinous cystic tumor complicated by an abscess caused by bacterial infection.

The adhesions between the ovarian tumor and ileum were manually dissected, and no fistula was observed between the two organs. At the adherent part of the ileum, all the layers of the ileal wall (approximately 40 cm along the long axis) were infiltrated with neutrophils, confirming acute peritonitis (Figure 2F–H). Acute peritonitis was found only in the ileum, sparing the other organs. The infiltrative shadow in the upper lobe of the right lung seen on the chest radiography and non‐contrast chest CT was not pneumonia but an adenocarcinoma (2.9 cm in diameter) (Figure 2I). There was a microscopic accumulation of neutrophils in the liver, suggestive of sepsis, but there were no histological findings of sepsis in the other organs. A few glomeruli remained in the kidney; however, the glomeruli were sclerotic, and the kidney vessels were thickened with fibrous intima, consistent with the histological findings of chronic kidney disease on dialysis.

## 3 DISCUSSION

There are no reviews on ovarian tumors with abscesses without fistulas to other organs, as reported here. We searched PubMed and found 10 similar cases, which are summarized in Table 1.^6^, ^7^, ^8^, ^9^, ^10^, ^11^, ^12^, ^13^, ^14^, ^15^ Of the 10 cases, there were seven cases of benign ovarian tumors (six cases of dermoid cyst and one case of mucinous cystadenoma) and three cases of malignant ovarian tumors (two cases of serous carcinoma and one case of squamous cell carcinoma derived from an ovarian dermoid cyst). All were surgical cases of ovarian tumors in patients with complaints of fever or abdominal pain (Table 1). In addition, obvious abscesses with fistulas to the gastrointestinal tract or other organs were found in eight cases, and in half of the cases, abscesses were detected preoperatively on imaging (presence of gas or an air‐fluid level in the tumor)^16^, ^17^, ^18^, ^19^, ^20^, ^21^, ^22^, ^23^ (Table 2).

**TABLE 1:** Cases of ovarian tumors with grossly visible abscesses without fistula to other organs reported in the past 30 years

	Author	Year	Age	Histology	Ovary diameter (cm)	Symptom	Microorganism
1	Hsueh	1992	75	Mucinous cystadenoma	35 × 30	Body weight loss	*Streptococcus agalactiae*
2	Yoshida	1993	70	Serous carcinoma	16	Fever, abdominal distension, and vaginal bleeding	*Escherichia coli*, *Pseudomonas aureginosa*, *Serratia marcescens*
3	Uwaydah	1998	37	Dermoid cyst	12 × 11 × 7	Fever	*Brucella melitensis*
4	Matsubayashi	2001	14	Dermoid cyst	12 × 10 × 8	Fever and abdominal discomfort	*Salmonella chester*
5	Khan	2005	77	Serous carcinoma	9 × 6 × 4	Fever and abdominal pain	*E. coli*
6	Luk	2007	20	Dermoid cyst	10 × 9.5	Fever and abdominal pain	*Chlamydia trachomatis*
7	Spencer	2011	14	Dermoid cyst	8.2 × 7 × 6.8	Fever and abdominal pain	*Methicillin‐sensitive Staphylococcus aureus*
8	Salehpour	2013	28	Dermoid cyst	10.1 × 4.2	Fever and abdominal pain	*Actinomyces* spp.
9	Chhabra	2018	8	Dermoid cyst	12 × 12 × 6.7	Fever and abdominal pain	*E. coli*
10	Bacalbasa	2020	47	Squamous cell carcinoma (SCC) derived from dermoid cyst	10 × 8 × 7	Abdominal pain	N/A
11	Our case	2022	82	Mucinous cystic tumor	24	Fever	*E. coli*, *Bacteroides vulgatus*, *Staphylococcus epidermidis*

Abbreviation: N/A, not applicable.

**TABLE 2:** Cases of ovarian tumors with grossly visible abscesses with fistula to other organs reported in the past 30 years

	Author	Year	Age	Histology	Ovary diameter (cm)	Symptom	Microorganism	Fistula	Preoperatively diagnosed as ovarian abscess on imaging
1	Protopapas	2004	78	Cyst adenocarcinoma	N/A	Vaginal bleeding and purulent secretion	N/A	Uterus	+
2	Upadhye	2005	48	Dermoid cyst	20 × 15	Fever and abdominal pain	*Salmonella Typhi*	Small intestine	−
3	Yahagi	2011	61	Clear cell carcinoma	26	Abdominal distension and bloody bowel discharge	N/A	Sigmoid	+
4	Song	2012	73	SCC derived from dermoid cyst	24	Abdominopelvic discomfort	N/A	Small intestine	−
5	Shai	2013	57	Serous carcinoma (high grade)	7.5	Fever and abdominal pain	*Escherichia coli*	Small intestine	+
6	Min	2015	67	SCC derived from dermoid cyst	9.8	Abdominal pain	N/A	Sigmoid	+
7	Yi	2015	42	Dermoid cyst	11 × 11	Abdominal pain	N/A	Rectosigmoid	−
8	Kizaki	2016	43	Dermoid cyst	11	Fever and diarrhea	N/A	Rectum	−

Abbreviation: N/A, not applicable.

In the 10 ovarian tumors without fistulas in Table 1, no causes of fever or abdominal pain other than the ovarian tumor were found. The ovarian tumors were surgically removed immediately. The inflammatory signs disappeared after surgery, signifying that the inflammatory signs were caused by an abscess within the ovarian tumor. However, in our case, because the chest X‐ray and non‐contrast chest CT showed shadows in the upper lobe of the right lung on admission, the inflammatory signs such as fever, increased white blood cell count, and elevated CRP were initially thought to be due to pneumonia. This pneumonia‐like imaging finding was a major obstacle in predicting the presence of an abscess within the ovarian tumor. If the patient had complained of abdominal pain, probably caused by the ovarian tumor, an oophorectomy would have been considered. Since the patient did not complain of abdominal pain, the possibility of an abscess within the ovarian tumor was not considered.

On admission, a non‐contrast abdominal CT was performed to assess the characteristics of the ovarian tumor, but there was no change in the contents of the ovarian tumor compared with the non‐contrast abdominal CT performed 15 months earlier. It was difficult to diagnose an ovarian abscess within the ovarian tumor from the non‐contrast abdominal CT scan because of the lack of gas or an air‐fluid level in the tumor. Since contrast‐enhanced abdominal CT had not been performed before admission, it may have been difficult to detect the ovarian abscess associated with the ovarian mucinous cystic tumor, even if contrast‐enhanced abdominal CT of the ovarian tumor had been performed at the time of admission.

A history of PID, intrauterine manipulation, or intrauterine contraceptive devices, which are well‐known causes of TOA, were absent in the patient. As shown in Figure 2, transmural inflammatory cell infiltration and acute peritonitis were observed in the ileum that was adherent to the ovarian tumor, but no inflammatory cells infiltration or acute peritonitis was observed in the other parts of the gastrointestinal tract not adherent to the ovarian tumor. The ileitis was probably caused by stagnation of the ileal contents and compression of the local circulation by the tumor. The spread of gastrointestinal inflammation (acute appendicitis, acute diverticulitis, etc.) to the ovary leading to ovarian abscess has been reported.^3^, ^4^, ^5^ Therefore, the ovarian abscess could be caused by the spread of bacteria from the ileitis in this case.

As shown in Table 1, the microorganisms causing ovarian abscesses are diverse, and more than one microorganism can be detected in an ovarian abscess. Therefore, even if an ovarian abscess can be diagnosed using imaging, it is difficult to determine an appropriate antibiotic. Currently, surgical resection appears to be the only treatment for ovarian abscesses.

Ovarian tumors complicated by abscesses are extremely rare, and the prevalence is unknown. This case suggests that abscesses may be associated with ovarian tumors. It is important to report similar cases which can be used to develop a method to detect abscesses in ovarian tumors by diagnostic imaging.

## AUTHOR CONTRIBUTIONS

All the authors were involved in the preparation of this manuscript. Daisuke Suzuki, Shiori Meguro, and Toshihide Iwashita analyzed all the pathological data and wrote the initial draft of the manuscript. Masahiro Hashimoto, Hideya Kawasaki, and Isao Kosugi analyzed and interpreted the clinical data. Koji Inagaki contributed to obtaining informed consent for the publication of this case report from the patient's family. Yasunori Enomoto, Miho Sugiyama, and Mayu Fukushima interpreted the data and critically revised the manuscript for intellectual content. All the authors have read and approved the final version of the manuscript.

## FUNDING INFORMATION

This work was supported by Grants‐in‐Aid for Scientific Research C (grant numbers 20K07370 and 21K06947) from the Japan Society for the Promotion of Science. The funder provided financial support for the study but was not involved in the design of the study, the collection, analysis, and interpretation of data, or the writing of the manuscript.

## CONFLICT OF INTEREST

The authors declare no conflicts of interest regarding the publication of this article.

## ETHICAL APPROVAL

This case report was approved by the Ethics Committee of the Chutouen General Hospital (approval number: CGH2022‐15).

## CONSENT

The written informed consent to publish the case details was obtained from the patient.

## Data Availability

The authors declare that all the relevant data have been included in this article and are available in this article.
